# On the optimal texture shape with the consideration of surface roughness

**DOI:** 10.1038/s41598-022-19094-8

**Published:** 2022-09-01

**Authors:** Guangyao Bei, Chenbo Ma, Xilong Wang, Jianjun Sun, Xingya Ni

**Affiliations:** College of Mechanical and Electronic Engineering, Nanjing Forestry University, Nanjing, 210037 China

**Keywords:** Mechanical engineering, Engineering

## Abstract

The optimal texture shape considering surface roughness is determined by solving the average Reynolds equation, selecting Jakobsson–Floberg–Olsson boundary conditions, and using a genetic algorithm. The effects of surface roughness as indicated by the combined root-mean-square (RMS), surface pattern parameter, and operating parameters on the friction coefficient, area ratio, and depth of the optimal self-defined shape and optimal dimple were studied. Results show that the friction coefficient will be significantly reduced during the shape optimization considering the effect of surface roughness. The variation laws of the optimal dimple area ratio with the combined RMS, surface pattern parameter, minimum film thickness, sliding speed and the variation law of the optimal depth of the optimal self-defined shape with surface roughness and working parameters are obtained. Finally, this study concludes that the influence of roughness parameters on the optimal dimple shape is greater than that on the optimal self-defined shape under different sliding speeds.

## Introduction

Surface texture technology has excellent performance in reducing friction and wear and improving bearing capacity. This technology can effectively reduce friction by machining regularly arranged pits or grooves on the bearing surface. Researchers have been concentrating their efforts in recent years on how to design the surface texture structure with the optimum friction performance.

In surface texture, the structural characteristics of a single texture can be described by its 3D shape, size, aspect ratio of texture, and other parameters. These parameters will affect the contact performance of surface texture. Accordingly, determining the best values of these parameters has become the main goal of many studies^1^. Malik et al.^2^ studied the dimensionless load-carrying capacity of full texture and partial texture sliding bearings, and found that when the aspect ratio is less than 1, the full texture has a higher load-carrying capacity, while the load-carrying capacity of partial texture is superior under the condition of higher aspect ratio. Li et al.^3^ processed V-grooves of cemented carbide with different texture density by femtosecond processing technology, and studied their tribological properties. The results showed that the herringbone texture with a certain texture density on the surface could help to reduce the friction on the surface of YT15 cemented carbide. Zum et al.^4^ made a survey on the friction properties of surface texture of ceramic materials. The results showed that the dimple, channel, width, depth, and area coverage of the surface microtexture would have a significant influence on the friction properties. Wang et al.^5^ did a research on the shape and structural parameters of surface texture of cylinder piston ring friction pair samples by orthogonal test. The results show that the friction characteristics of materials can be promoted by deepening the texture depth, increasing the surface density and size. Wang et al.^6^ did a research on the influences of geometric parameters of texture unit on tribological behavior by making annular surface texture on AISI1045 steel surface. The results show that in the annular texture, changing the width will considerably scale back the friction, and the optimal texture width with the smallest friction is obtained. Krupka et al.^7^ studied the influence of microdents within thin elastohydrodynamics (EHD) contact and found that the depth of microdents has a very important impact on the performance of a lubricating film. While the micro dent depth of the surface texture is in a medium degree, the lubrication performance of the lubricating film can be effectively improved. Ryk et al.^8^ considered the effect of texture depth on tribological properties, and studied it under the condition of oil starvation by experimental method. They found that there will be an optimal dimple depth for minimizing friction regardless of the viscosity of the oil. Yan et al.^9^ concentrated on the impact of structural parameters of surface texture dimple on the friction characteristics of sliding surface with orthogonal experiment method. Through the analysis of range and variance, the area ratio of the dimple has the main impact on the friction coefficient.

The operating condition parameters additionally influence the contact properties of surface, in addition to the structural parameters. Under the mixed lubrication condition, the characteristics of fractal-like texture surface is studied by Wang et al.^10^. The results showed that while the sliding speed increments, the friction coefficient of textured surface decreases. Khonsari et al.^11^ studied the sealing structure with a dimple surface texture and the capability of thrust bearing. They supposed that that there is an ideal optimal depth or depth diameter magnitude relation which will offer the most bearing capability, and the optimal depth will increment with the operating speed. Yu et al.^12^ considered the impact of dimple shapes on the tribological properties of surface and found that the antifriction effect decreased with the increase in experimental load, regardless of the dimple shape, and the differences between dimple shapes also became smaller. Yu et al.^13^ concentrated on the impact of liquid speed and load on the surface texture dimple pattern. The surface texture pattern designed according to the hydrodynamic principle has excellent tribological performance under the state of high velocity and low load. Meanwhile, the friction reduction effect of shallow and small dimples is better at low velocity and high load.

The texture shape will also influence the fundamental capacity of surface texture by improving the hydrodynamic bearing capacity^14^. Many studies have explored the tribological properties of dimples with different shapes through numerical simulation and experiments. Zhan et al.^15^ utilized nanosecond laser to generate six forms of surface texture including monomorphic texture and polymorphic texture on 40Cr steel, and carried out reciprocating sliding test under dry friction condition. The results show that different texture forms will produce an effect on the tribological properties. Uddin MS et al.^16^ studied the influences of geometric parameters such as texture shape, bottom contour, direction and depth of texture on the surface friction characteristics of the hydrodynamic slider. The square with a solitary wedge base shape has good tribological properties. For triangular texture, V-texture and elliptical texture, the height of the sliding direction affects their performance. For dimple texture patterns with different directions, Wang et al.^17^ studied the average hydrodynamic pressure produced by them, like circle, ellipse, and triangle in the sliding direction, based on the single dimple theoretical model. The result shows that the section shape with the highest loading capacity is the conical section placed perpendicular to the sliding direction when some structural parameters of surface texture are the same. Qiu et al.^18^ compared the bearing capacity of six air lubricated textured parallel bearings with diverse pit shapes, concluded that the ellipsoidal pit has greater bearing capacity, and proposed that the operating conditions can not affect the optimal geometry of surface texture. Wu et al.^19^ considered the partial shape dependence problem and the basis vector method, and proposed the basic vector method to optimize the design of complex shapes.Shen et al.^20^ considered the ideal texture shape with the highest bearing capability, and obtained the optimal texture shape below one-way and two-way slippery conditions based on the sequential quadratic programming algorithm, and compared the performance of optimal texture and regular texture. In order to obtain the true morphology of surface texture more accurately, many researchers have studied the computer generation of surface texture. To solve the mechanical filtering effect caused by the radius of the stylus tip when the contact stylus instrument measures the surface texture, Yoshida et al.^21^ used fast Fourier transform to study the amplitude transmission characteristics caused by the tip of the cosine wave stylus, and checked the wavelength limit. Uchidate et al.^22^ proposed a program to generate 3D random terrain data sets with periodic boundaries, which can avoid edge effects by assuming periodic boundaries, and evaluate the original data of surface metrology algorithms and measurement standards. Yang et al.^23^ studied the contact between rigid plane space and Gaussian isotropic elastic rough surface by using the methods of numerical generation and conjugate gradient fast Fourier transform, and obtained the seepage threshold based on the contact state.

The influence of surface roughness is not considered in most current research on texture optimization^24^. However, the texture of surface texture is made on top of the machined surface. In reality, there is no ideal smooth surface. Considering surface roughness is necessary in studying the texture optimization and friction lubrication characteristics of a surface texture. The affect of surface roughness on the friction properties of texture is studied by Zhou et al.^25^. They found an optimal friction value, which can make the lubricating film have the maximum bearing capacity. It is proposed that the bearing capacity of a lubricating film will be decided by surface texture, surface roughness, and their interactivity. Ma et al.^26^. conducted friction experiments on a silicone rubber and GCr15 bearing steel balls with different surface roughness and texture. They proposed that the surface roughness of the sample has an optimal range that can optimize the friction performance of the sample when the lubrication state of the friction pair is in the mixed condition. Mechanical seals and thrust bearings exist on complex textured surfaces, in order to predict their friction performance, based on finite element, an average flow model that can maintain mass conservation is established by Xie et al.^27^. They proposed that surface roughness can enhance the beneficial and adverse impact of dimples of different shapes. Furthermore, the influence of surface roughness becomes more and more obvious under the condition of the ratio of nominal film thickness to comprehensive roughness decreases.

Using the method of average Reynolds equation and combining genetic algorithm, the optimal texture shape considering surface roughness is studied in this study. For purpose of considering the cavitation effect, the Jakobsson–Floberg–Olsson mass-conservation boundary condition is considered. Furthermore, the influence of roughness, surface pattern, and operating parameters on the coefficient of friction, area ratio, and depth of the optimal self-defined shape and the optimal dimple is analyzed.

## Materials and methods

### Geometric model

**Figure 1:**
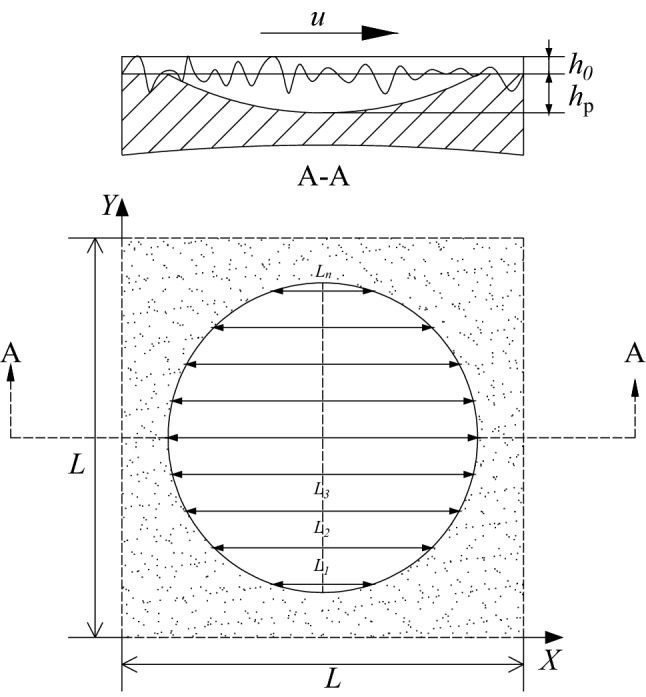
Geometrical model for texture shape optimization considering surface roughness (a unit cell with one dimple).

**Figure 2:**
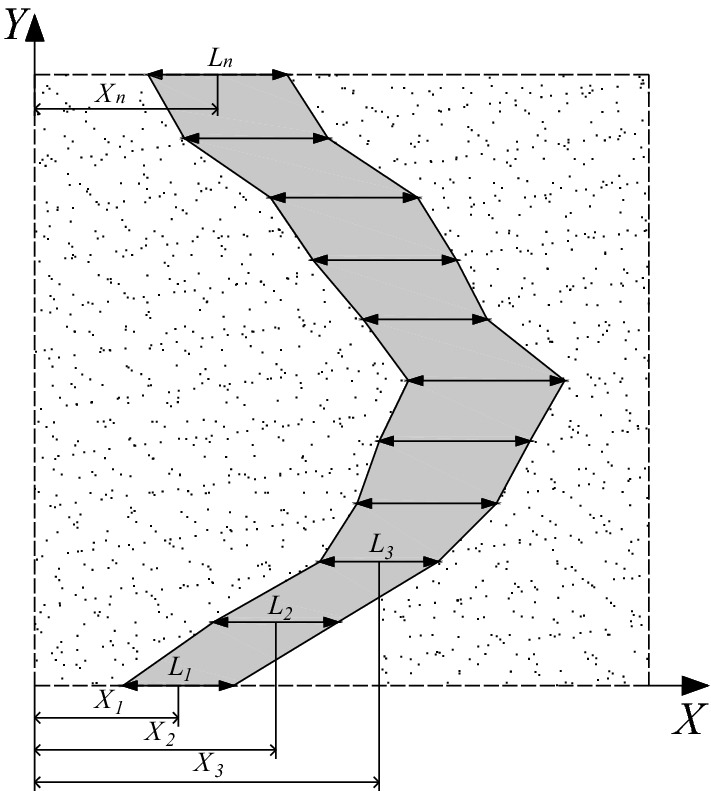
Geometrical model for texture shape optimization considering surface roughness (design variables for an arbitrary texture shape).

The geometric model of single dimple optimization considering surface roughness, as described in reference^20^ is showed in Fig. 1. The calculation domain is a square element containing a single dimple, and the impact of texture and roughness is considered. *U* indicates the speed of the runner surface and moves along the X-axis of the stationary textured surface. Figure 2 shows an optimized geometric model of any texture shape considering surface roughness. Construct multiple horizontal lines in the computational domain whose length (from *L*_1_ to *L*_n_) and center position (from *X*_1_ to *X*_n_) can be set by themselves. By connecting them, any texture shape can be constructed.

### Governing equation and objective function

Patir and Cheng^28^ came up with an expression of the average Reynolds equation as following, which considers the roughness effect and is a two-dimensional steady-state form of the laminar Reynolds equation of incompressible Newtonian fluid.

1 $$\frac{\partial }{\partial x}\left( {\phi_{x} h^{3} \frac{{\partial \overline{p} }}{\partial x}} \right) + \frac{\partial }{\partial z}\left( {\phi_{z} h^{3} \frac{{\partial \overline{p} }}{\partial z}} \right) = 6\mu u\left( {\frac{{\partial \overline{{h_{T} }} }}{\partial x} + \sigma \frac{{\partial \phi_{s} }}{\partial x}} \right)$$

The connection between $\partial \overline{h}_{T} /\partial x$ and $\partial h/\partial x$ is capable of developing with Eq. (1) by introducing a dimensionless parameter *ϕ*_c_ called contact coefficient^29^ , so Eq. (1) can be expressed as:

2 $$\frac{\partial }{\partial x}\left( {\phi_{x} h^{3} \frac{{\partial \overline{p} }}{\partial x}} \right) + \frac{\partial }{\partial z}\left( {\phi_{z} h^{3} \frac{{\partial \overline{p} }}{\partial z}} \right) = 6\mu u\left( {\phi_{c} \frac{\partial h}{{\partial x}} + \sigma \frac{{\partial \phi_{s} }}{\partial x}} \right).$$

The surface pattern parameter is represented by *γ*^28–30^ , which determine the values of flow and contact coefficient in Eq. (2) and *h*/*σ* repressents the ratio of nominal film thickness to comprehensive RMS roughness. For the dimple in this study in Fig. 1, the nominal film thickness *h* is able to be defined in this way:

3 $$h = \left\{ {\begin{array}{*{20}l} {h_{0} } & {{\text{outside}}\;{\text{the}}\;{\text{shape}}} \\ {h_{0} + h_{p} } & {{\text{inside}}\;{\text{the}}\;{\text{shape}}} \\ \end{array} } \right..$$

On behalf of predicting the cavitation behavior, the JFO cavitation theory realized by mass conservation algorithm is adopted in this study, and by setting the "predetermined" cavitation pressure *p*_cav_, it is ensured that the pressure in the cavitation area remains unchanged. According to the JFO theory proposed by Elord and Adams^31^, Eq. (2) is able to be described as the following form^32^:

4 $$\frac{\partial }{\partial x}\left( {\phi_{x} h^{3} \frac{{\partial \overline{p} }}{\partial x}} \right) + \frac{\partial }{\partial z}\left( {\phi_{z} h^{3} \frac{{\partial \overline{p} }}{\partial z}} \right) = 6\mu u\left( {\phi_{c} \frac{\partial \theta h}{{\partial x}} + \sigma \frac{{\partial \theta \phi_{s} }}{\partial x}} \right),$$

where *θ* indicates the film content, and when *p* ≥ *p*_cav_, the value of *θ* is 1, when *p* = *p*_cav_, the value of *θ* is 0.

The average pressure distribution in Eq. (4) is solved with the method of continuous over relaxation Gauss–Seidel iterative.

With regard to the contact pressure *p*_c_, Greenwood and Tripp^33^ developed the rough surface contact model used in this study:

5 $$p_{c} = K^{^{\prime}} E^{*} F_{2.5} \left( {{h \mathord{\left/ {\vphantom {h \sigma }} \right. \kern-\nulldelimiterspace} \sigma }} \right),$$

where the equivalent elastic modulus is expressed by E^*^, and *K’* is a constant in the contact pressure equation, which is taken as 5.319 × 10^10^ × σ^2.5^^34^ in this study, and the probability distribution of roughness height is represented by *F*_2.5_(*h*/σ), which is able to be described as:

6 $$F_{2.5} \left( {{h \mathord{\left/ {\vphantom {h \sigma }} \right. \kern-\nulldelimiterspace} \sigma }} \right) = \left\{ {\begin{array}{*{20}l} {B\left( {\Omega - {h \mathord{\left/ {\vphantom {h \sigma }} \right. \kern-\nulldelimiterspace} \sigma }} \right)^{Z} } \hfill & {{h \mathord{\left/ {\vphantom {h {\sigma \le \Omega }}} \right. \kern-\nulldelimiterspace} {\sigma \le \Omega }}} \hfill \\ 0 \hfill & {h/\sigma > \Omega } \hfill \\ \end{array} } \right..$$

After calculating the average pressure distribution and rough surface contact pressure, the average pressure of the unit cell represented by *W*_avg_ and the bearing capacity represented by *W* can be expressed as:

7 $$W_{{{\text{avg}}}} = W{/}L^{2} ,$$

8 $$W = \int_{{ - \frac{L}{2}}}^{\frac{L}{2}} {\int_{{ - \frac{L}{2}}}^{\frac{L}{2}} {\left( {\overline{p} + p_{c} } \right)} } {\text{d}}x{\text{d}}z.$$

Dry friction will occur in the contact between micro convex bodies, and viscous shear stress will produce viscous friction in the whole oil film area. The sum of the two constitutes the friction *F*^30^

9 $$F = \int_{{ - \frac{L}{2}}}^{\frac{L}{2}} {\int_{{ - \frac{L}{2}}}^{\frac{L}{2}} {\left[ {\frac{\mu u}{h}\left( {\phi_{f} - \phi_{fs} } \right) + \mu_{f} p_{c} } \right]} } {\text{d}}x{\text{d}}z.$$

Thus, the friction coefficient of the friction pair is obtained at last

10 $$f = F{/}W.$$

### Solution method

Genetic algorithm is used to solve the optimal texture shape of surface texture in this study, because genetic algorithm can effectively solve the optimization problems of nonlinearity and constraints. Genetic algorithm is a probabilistic optimization method. It can carry out effective global search, search all the solutions in the solution space quickly, and make use of its internal parallelism to facilitate distributed computing and accelerate the solution speed. This algorithm has good convergence and robustness compared with some traditional optimization methods and has less calculation time under the condition of required calculation accuracy. Moreover, this algorithm does not depend on the setting of the initial value and is not easy to fall into local solution compared with the SQP method in reference^20^. First, set the friction coefficient as the objective function of genetic algorithm optimization, set the crossover operator as 0.8, set the mutation operator as 0.2, set the population number as the default value, set the maximum number of iterations as 500, set the center position and length of the optimized texture as the design variables, and set the end threshold as 1 × 10^–6^. In order to make the optimized target texture in the computational domain, inequality constraints are added to the genetic algorithm as follows:

11 $$X_{i} - 0.5 \times L_{i} \ge 0$$

12 $$X_{i} + 0.5 \times L_{i} \le 0$$

The results of each optimization are different. Each group of data is calculated more than three times, and the maximum value is used as the simulation value.

### Model validation

The input parameter values simulated using the SQP method are illustrated in Table 1, and the input parameter values of simulation using genetic algorithm under the same conditions are illustrated in Table 2.

**Table 1:** Input parameters for simulation I.

Parameter	Value
Unit cell length *L* (mm)	5
Minimum film thickness *h*_0_ (μm)	10
Viscosity *μ* (Pa s)	0.038
Ambient pressure *p*_0_ (Pa)	1 × 10^5^
Sliding speed *U* (m/s)	1

**Table 2:** Input parameters for simulation II.

Parameter	Value
Unit cell length *L* (mm)	5
Minimum film thickness *h*_0_ (μm)	4
Viscosity *μ* (Pa s)	0.038
Area ratio of texture	30%
Cavitation pressure (Pa)	0.3 × 10^5^
Ambient pressure *p*_0_ (Pa)	1 × 10^5^
Sliding speed *U* (m/s)	0.01–5.12

Table 3 shows the numerical comparison between the simulation results in this study and reference^20^.

**Table 3:** Dimensionless design variables and the corresponding ${\overline{\text{W}}}$ in reference^17^ and this study.

	$\backslash$	$\overline{L}_{1}$	$\overline{X}_{2}$	$\overline{L}_{2}$	$\overline{X}_{3}$	$\overline{L}_{3}$	$\overline{X}_{4}$	$\overline{L}_{4}$	$\overline{X}_{5}$	$\overline{L}_{5}$	$\overline{X}_{6}$	$\overline{L}_{6}$	$\overline{X}_{7}$	$\overline{L}_{7}$	$\overline{h}_{g}$	${\overline{\text{W}}}$	Area ratio (%)
**Texture shape without area ratio limitation**																	
Ref.^17^	0.10	0.18	0.38	0.48	0.66	0.66	0.75	0.48	0.66	0.66	0.38	0.48	0.10	0.18	1.63	38.26	49.0
This study	0.04	0.15	0.45	0.68	0.70	0.78	0.91	0.88	0.76	0.73	0.38	0.48	0.05	0.21	1.82	40.47	55.0
**Texture shape with area ratio of 30%**																	
Ref.^17^	0.08	0.14	0.39	0.46	0.72	0.54	0.94	0.10	0.88	0.22	0.46	0.40	0.09	0.16	18.6	36.12	30.0
This study	0.07	0.17	0.39	0.56	0.75	0.27	0.89	0.19	0.95	0.18	0.44	0.43	0.05	0.15	19.9	37.15	30.0

**Figure 3:**
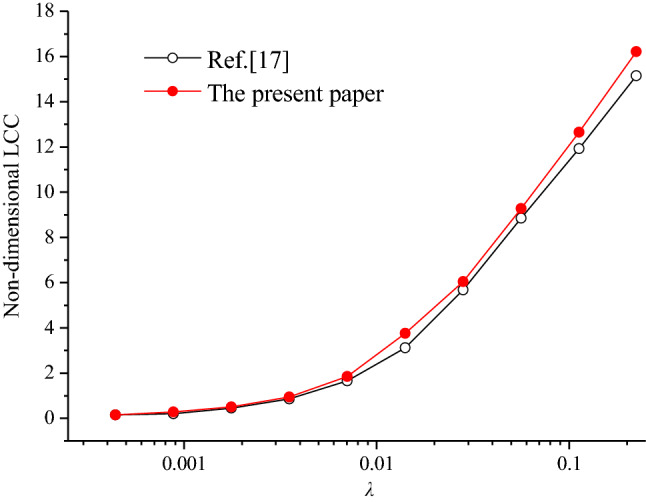
Comparison of the LCCs of optimum directional shapes between this study and reference^17^.

Figure 3 shows the LCCs of optimum directional shapes under different λ values in this paper and reference^20^. The simulated operating conditions are represented by a dimensionless parameter λ is in direct proportion to the reciprocal of lubricant viscosity μ, sliding speed U and bulk modulus β. Figure 3 depicts that the simulation results in this study are basically the same as those in reference^20^. Moreover, the values of the LCCs of optimum directional shapes in this study are larger than those in reference^20^ under different simulation parameters. The main reason is that genetic algorithm, which is a better optimization algorithm, is adopted in this study. Genetic algorithm can obtain better texture shape and bearing capacity compared with the SQP method, which easily falls into the local optimal solution depending on the position of the starting point adopted in reference^20^, because it has the advantage of global optimization.

The JFO condition meeting the mass conservation should be adopted because the Half-Sommerfield condition used in the bearing capacity calculation in reference^20^ does not meet the mass conservation condition, which is quite different from the actual situation under many conditions.

Tables 4 and 5 show the texture shape parameters and dimensionless bearing capacity while the texture area ratio is 30% and unrestricted. The table shows that the bearing capacity under Half-Sommerfield condition is greater than that under JFO condition, mainly due to Half-Sommerfield condition overestimates the value of cavitation area. The numerical results showed that the differences between the two conditions can reach more than 15%. The differences may further increase, especially with the change of working condition parameters. Therefore, the JFO cavitation boundary condition must be applied in texture shape optimization. On this basis, the follow-up research of this study is carried out under the JFO condition.

**Table 4:** Dimensionless design variables and the corresponding cavitation area ratio and ${\overline{\text{W}}}$ for the JFO and Half-Sommerfield conditions (area ratio = 30%).

	$\overline{X}_{1}$	$\overline{L}_{1}$	$\overline{X}_{2}$	$\overline{L}_{2}$	$\overline{X}_{3}$	$\overline{L}_{3}$	$\overline{X}_{4}$	$\overline{L}_{4}$	$\overline{X}_{5}$	$\overline{L}_{5}$	$\overline{X}_{6}$	$\overline{L}_{6}$	$\overline{X}_{7}$	$\overline{L}_{7}$	$\overline{h}_{g}$	${\overline{\text{W}}}$	Area ratio (%)	Cavitation area ratio (%)
JFO	0.10	0.20	0.51	0.33	0.83	0.43	0.95	0.19	0.88	0.24	0.52	0.39	0.02	0.21	0.48	8.76	30.0	1.2
Half-Summerfield	0.12	0.20	0.63	0.15	0.99	0.11	0.96	0.14	0.69	0.62	0.37	0.62	0.02	0.13	0.42	9.32	30.0	2.8

**Table 5:** Dimensionless design variable and the corresponding cavitation area and ${\overline{\text{W}}}$ for the JFO and Half-Sommerfield conditions (unrestricted).

	$\overline{X}_{1}$	$\overline{L}_{1}$	$\overline{X}_{2}$	$\overline{L}_{2}$	$\overline{X}_{3}$	$\overline{L}_{3}$	$\overline{X}_{4}$	$\overline{L}_{4}$	$\overline{X}_{5}$	$\overline{L}_{5}$	$\overline{X}_{6}$	$\overline{L}_{6}$	$\overline{X}_{7}$	$\overline{L}_{7}$	$\overline{h}_{g}$	${\overline{\text{W}}}$	Area ratio (%)	Cavitation area ratio (%)
JFO	0.02	0.33	0.44	0.35	0.92	0.86	0.83	0.43	0.99	0.93	0.51	0.42	0.06	0.21	0.46	9.13	42.6	0.0
Half-Summerfield	0.02	0.10	0.39	0.64	0.66	0.73	0.85	0.70	0.71	0.72	0.43	0.54	0.08	0.18	0.35	10.76	54.3	4.9

### Parameter setting

Table 6 illustrates some basic parameter settings used in the simulation of this study.

**Table 6:** Input parameters for the simulation.

Parameter	Value
Combined RMS σ (μm)	0.05–2
Surface pattern parameter γ	1/6–6
Sliding velocity *u* (m/s)	0.05–4
Minimum film thickness *h*_0_ (μm)	1–5
Cavitation pressure *P*_cav_ (kPa)	0–90

## Results and discussion

### Impact of roughness parameters on the coefficient of friction, area ratio, and optimal depth

**Figure 4:**
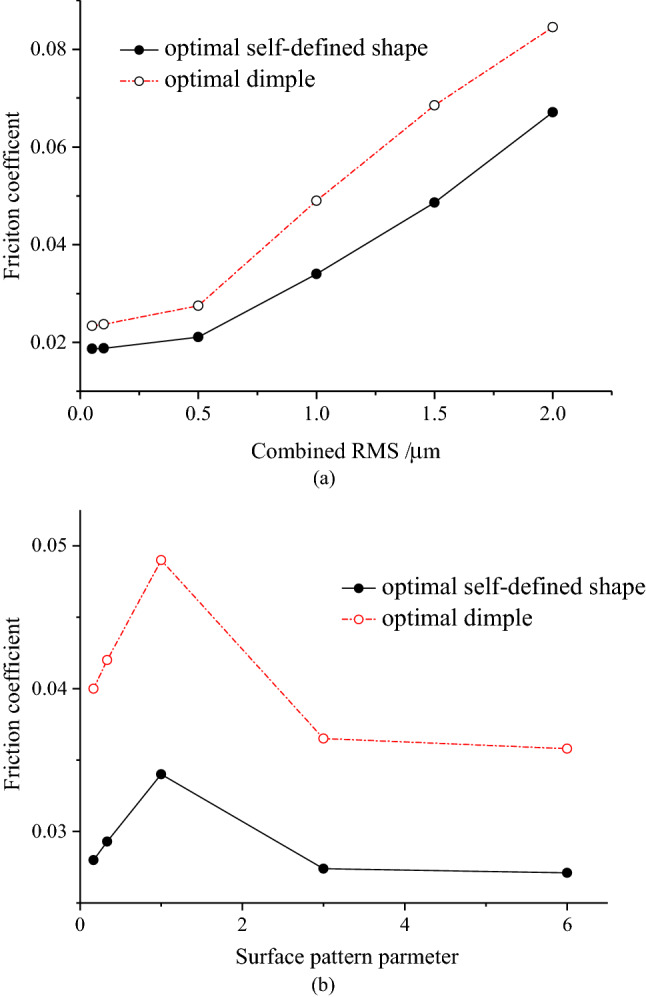
(**a**) Variation of the optimal friction coefficient with combined RMS; (**b**) Variation of the optimal friction coefficient with surface pattern parameter.

The variation of friction coefficient with combined RMS for optimal self-defined shape and optimal dimple is depicted in Fig. 4a, and the variation of friction coefficient with surface pattern parameter is depicted in Fig. 4b. As the combined RMS for the optimal self-defined shape and optimal dimple is increased, the friction coefficient increases. Specifically, the friction coefficient slowly increases when the combined RMS is less than 0.5 μm (*h*_0_/*σ* < 4) and then evidently increases. Moreover, the friction coefficient of the optimal self-defined shape is more than 20% less than that of the optimal dimple under different combined RMS, indicating the necessity of shape optimization. Meanwhile, the friction coefficients of the optimal self-defined shape and the optimal dimple increase first and then decrease with the increases in the surface pattern parameter. The differences between the minimum and the maximum friction coefficients are 25.5% and 36.9%, respectively, indicating the importance of considering the surface pattern parameter. In addition, the friction coefficient of the optimal self-defined shape is more than 24% smaller than that of the optimal dimple under different surface pattern parameters, indicating the necessity of carrying out shape optimization research.

The reason why the optimal self-defined shape is better than the optimal dimple shape is that its shape can better comply with the flow direction, produce greater oil film loading capacity and smaller coefficient of friction. In order to further clarify its mechanism, this study further analyzes the area ratio and depth of the optimal self-defined shape and the optimal dimple shape.

**Figure 5:**
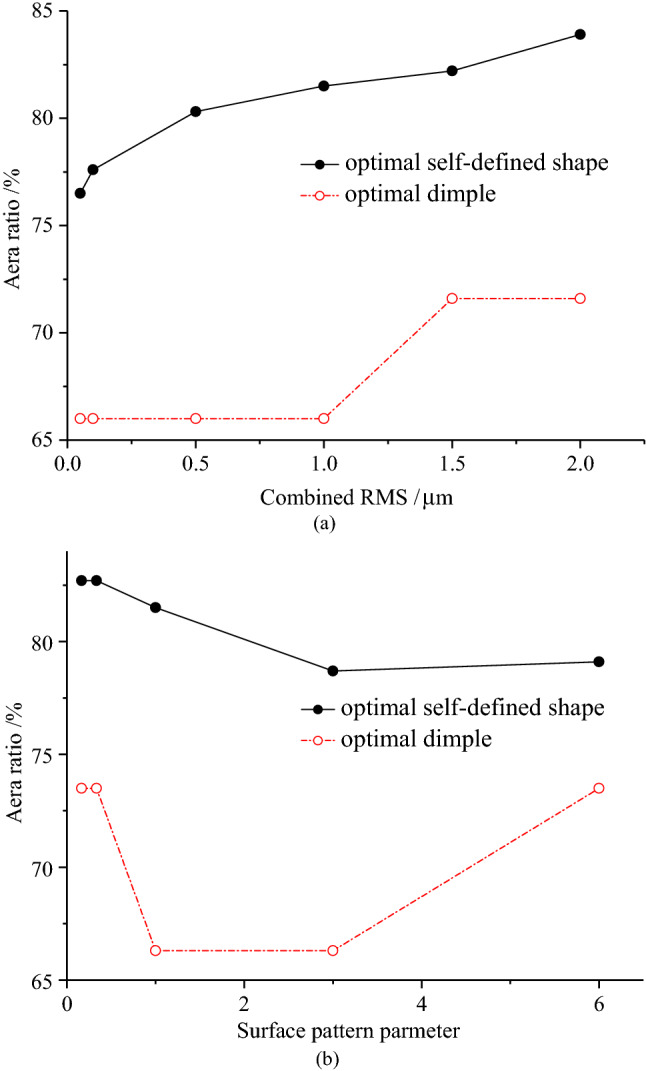
(**a**) Variation of the optimal area ratio with combined RMS; (**b**) variation of the optimal area ratio with surface pattern parameter.

Figure 5a shows the area ratios of the optimal self-defined shape and optimal dimple with combined RMS, and Fig. 5b shows the area ratios of the optimal self-defined shape and optimal dimple with surface pattern parameter is presented. The area ratio of the optimal self-defined shape is larger than that of the optimal dimple, which may be the reason for the smaller friction coefficient under the optimal self-defined shape. Moreover, the area ratio of the optimal self-defined shape increases with the increase in combined RMS, but as surface pattern parameter increases, the area ratio of the optimal self-defined shape firstly decreases and then increases. For the optimal dimple area ratio, there are two values, which are 66.0% and 71.6%, and its total change trends with the combined RMS and surface pattern parameter are the same as those under the optimal self-defined shape.

**Figure 6:**
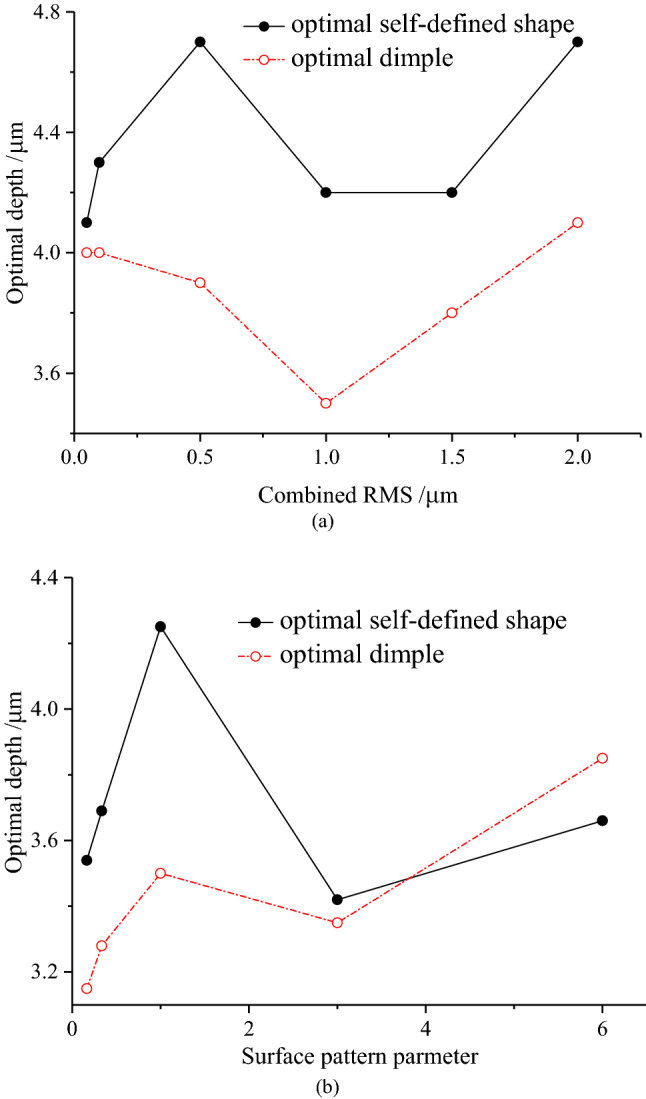
(**a**) Variation of the optimal depth with combined RMS; (**b**) variation of the optimal depth with surface pattern parameter.

Figure 6a shows the optimal depth of the optimal self-defined shape and optimal dimple with combined RMS, and Fig. 6b shows the optimal depth of the optimal self-defined shape and optimal dimple with surface pattern parameter. Furthermore, the roughness parameters have little influence on the optimal depth, which ranges from 4.1 to 4.7 μm and 3.5 μm to 4.2 μm, under different combined RMS and from 3.5 to 4.2 μm and 3.1 μm to 3.8 μm under different surface pattern parameters for the optimal self-defined shape and optimal dimple, respectively.

#### Variation of the friction coefficient, area ratio, and optimal depth with operating parameters

1. Variation of friction coefficient

**Figure 7:**
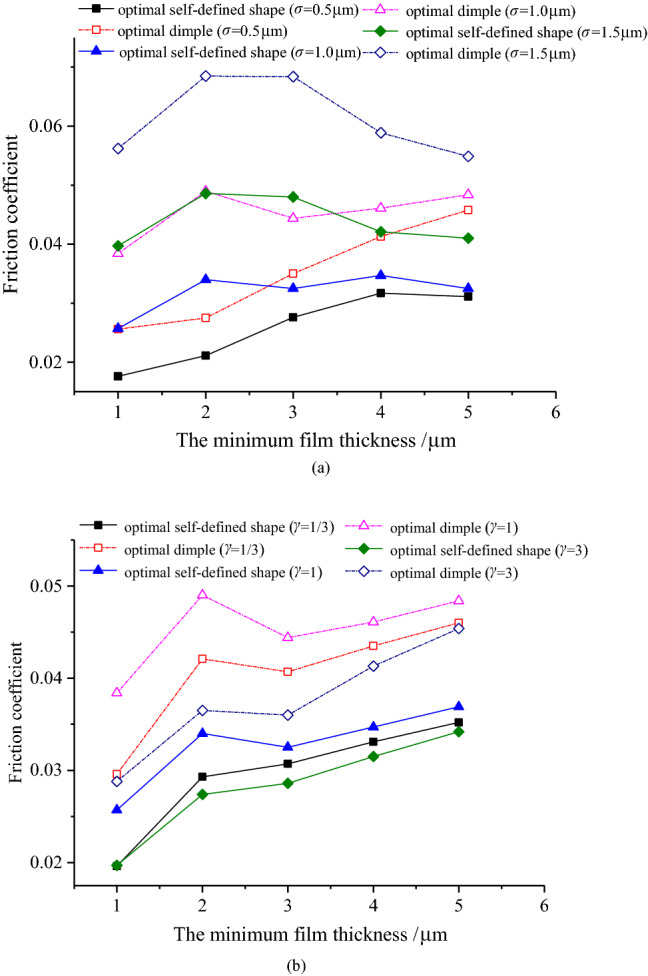
Variation of the friction coefficient with the minimum film thickness.

For the optimal self-defined shape and optimal dimple under different roughness parameters, their friction coefficients have the identical law with the minimum oil film thickness, which is illustrated in Fig. 7. Specifically, under the condition of the minimum film thickness increases, the coefficient of friction gradually increases (when the combined RMS is small) and then decreases (when the combined RMS is large). Moreover, with different surface morphology parameters, under the condition of the minimum film thickness increases, the friction coefficient increases gradually and reaches the maximum at *γ* = 1. Furthermore, the friction coefficient under the optimal self-defined shape under different roughness parameters is less than that under the optimal dimple, and this phenomenon is more obvious while the minimum film thickness is relatively small.

**Figure 8:**
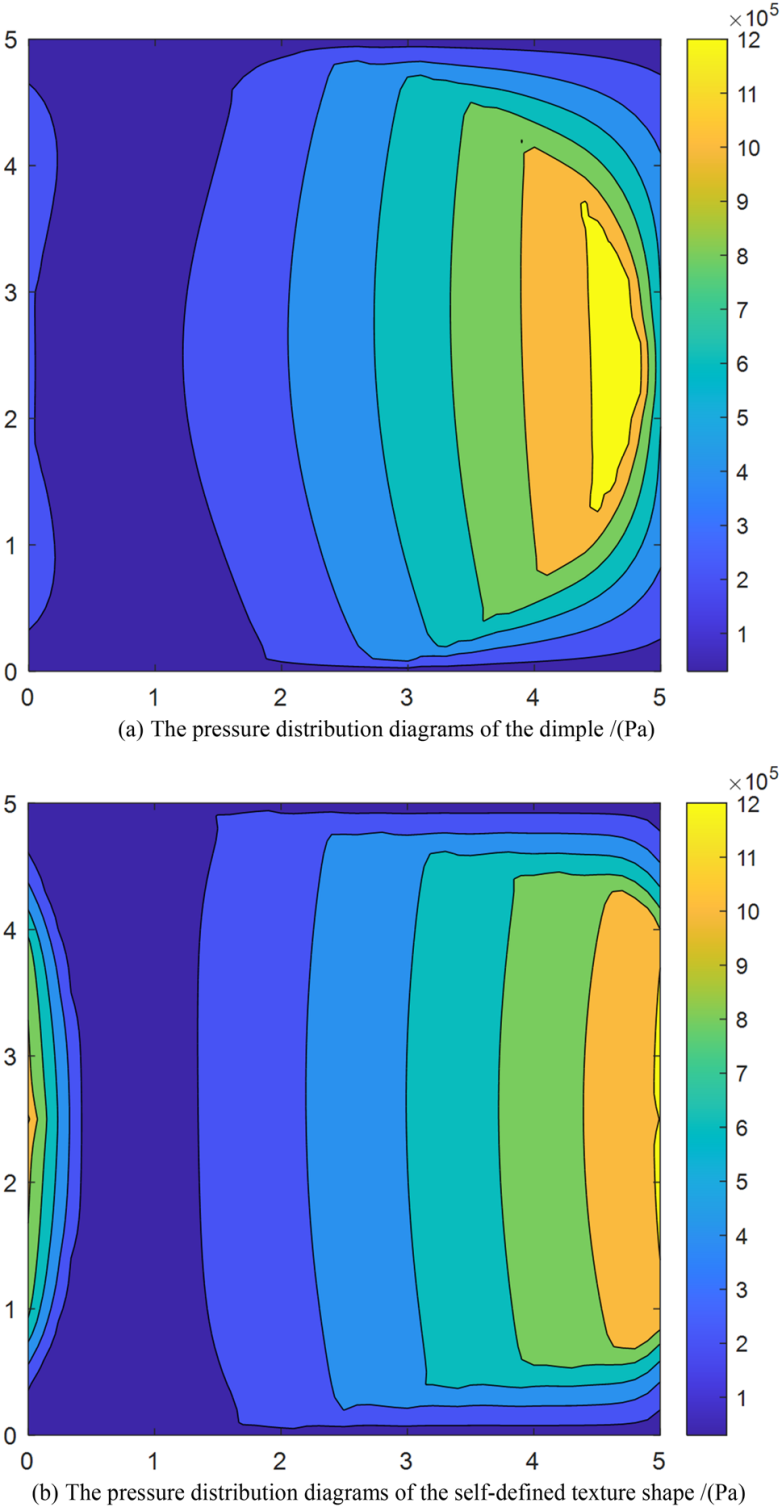
The pressure distribution of the dimple and the self-defined texture shape.

**Figure 9:**
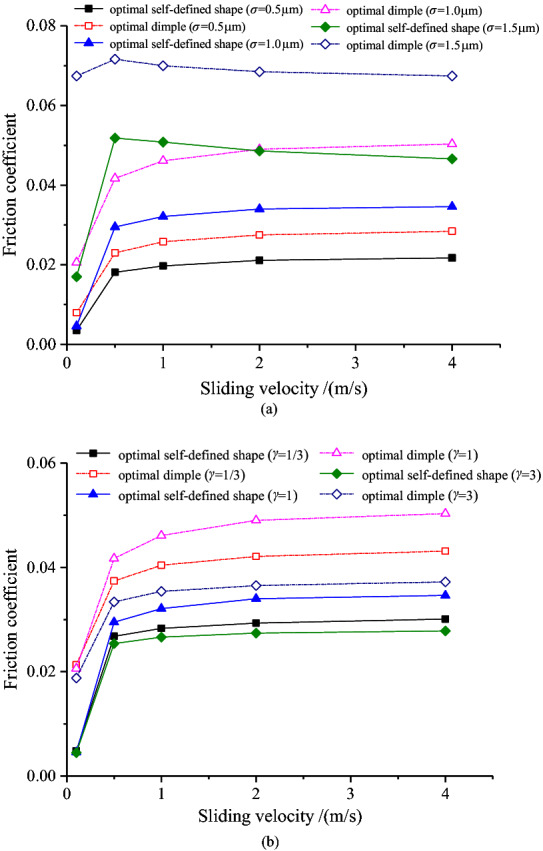
Variation of the friction coefficient with sliding velocity.

Figure 8 shows the pressure distribution of the dimple and the self-defined texture shape when the sliding speed is 1 m/s. Regardless of the sliding velocity and roughness parameter settings, the optimal self-defined shape has a smaller coefficient of friction for the optimal self-defined shape and optimal dimple, as presented in Fig. 9. In the sliding velocity under the condition of *u* is less than 0.5 m/s, the increase of sliding velocity will significantly increase the coefficient of friction, and the influence of the surface pattern parameter is small. When the sliding velocity is large, it has little impact on the coefficient of friction. Furthermore, the influence of the roughness parameters on the optimal dimple is greater than that on the optimal self-defined shape under different sliding velocities.

**Figure 10:**
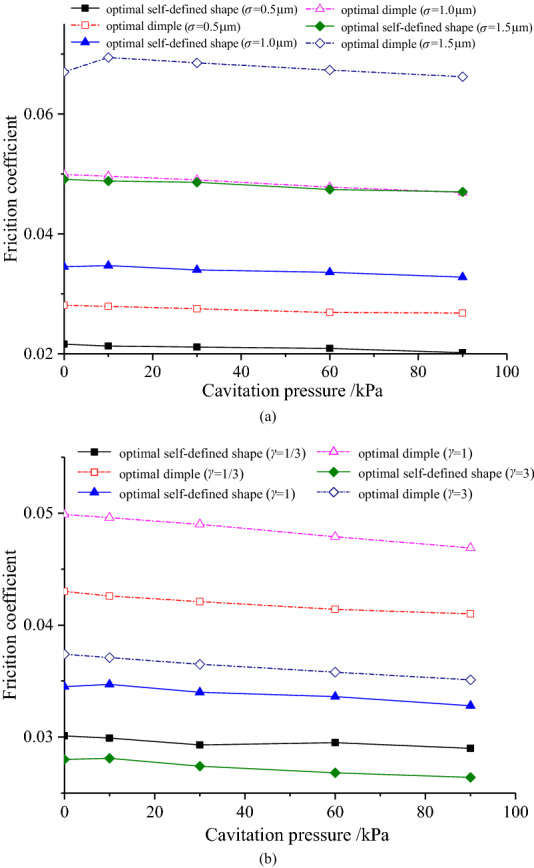
Variation of the friction coefficient with cavitation pressure.

Figure 10 shows that when the roughness conditions are diverse, even if the cavitation pressure changes, the change of friction coefficient is very small. By analyzing the change of cavitation area ratio, it can be found that cavitation will hardly occur under the design conditions. There will only be a small part of the cavitation area exists when the cavitation pressure is less than 30 kPa, but all of them are less than 2%. This situation should be the reason why the effect of cavitation pressure is not evident.

(2) Variation of area ratio

**Figure 11:**
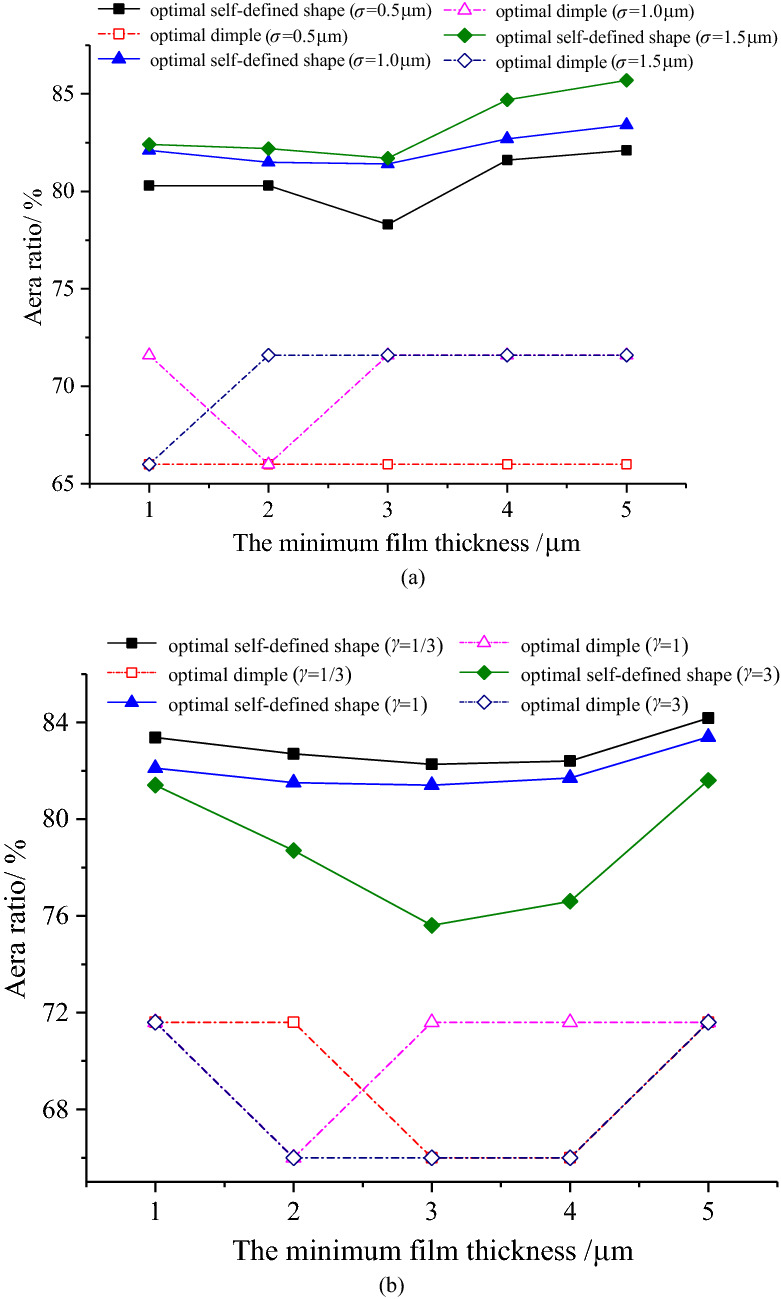
Variation of the area ratio with the minimum film thickness.

**Figure 12:**
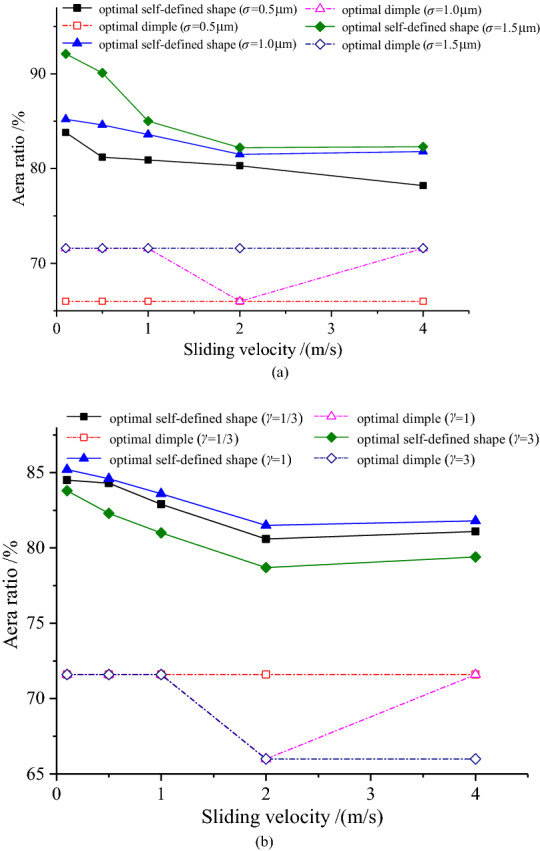
Variation of the area ratio with sliding velocity.

**Figure 13:**
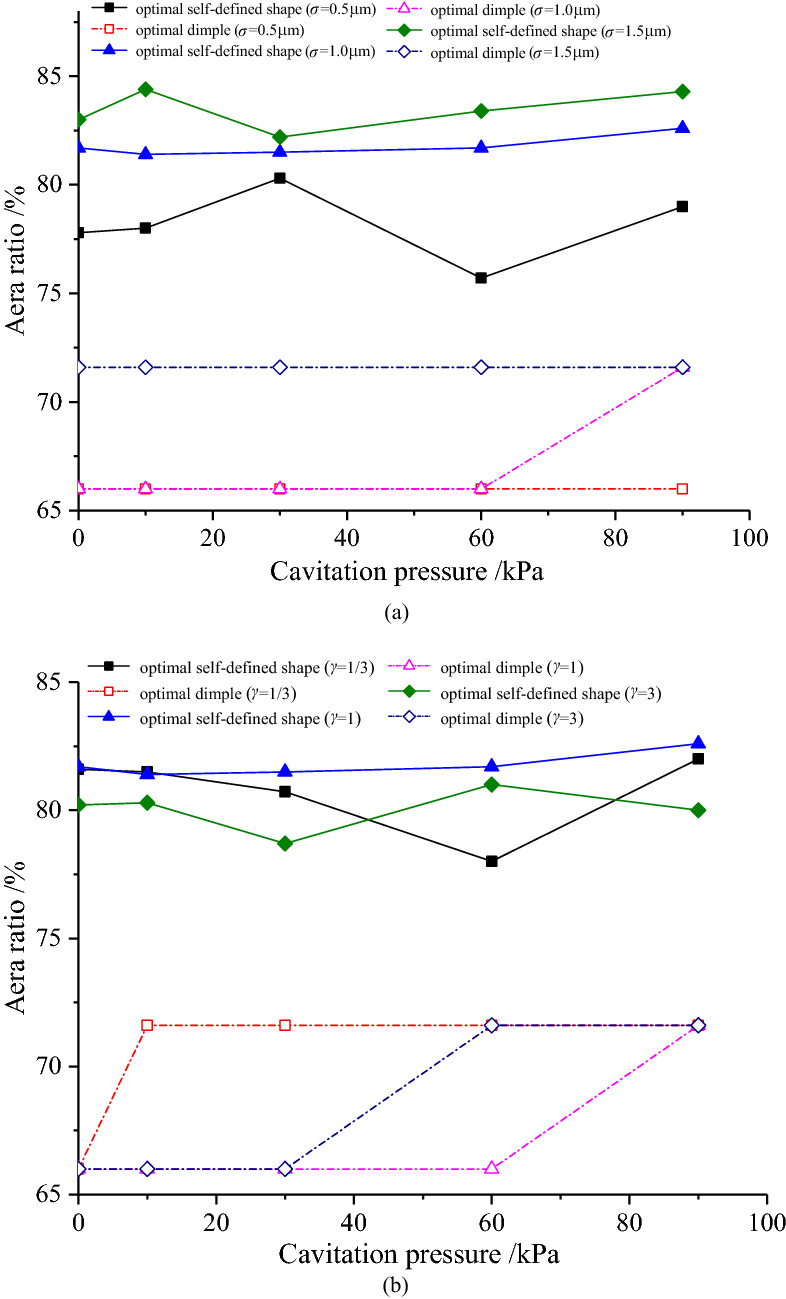
Variation of the area ratio with cavitation pressure.

Figures 11, 12 and 13 shows the area ratio of the optimal self-defined shape is greater than that of the optimal dimple under various operating parameters. Specifically, under the condition of the minimum film thickness increases, the area ratio of the optimal self-defined shape decreases firstly and then increases, as shown in Fig. 11. Furthermore, the larger the combined RMS and the smaller the surface pattern parameter, the larger the area ratio becomes.

As the sliding velocity increases, the area ratio of the optimal custom shape firstly decreases and then increases slowly, and the area ratio increases as the combined RMS increases, which are shown in Fig. 12. In addition, the area ratio reaches the maximum at *γ* = 1 under different sliding velocities.

The area ratio of the optimal self-defined shape fluctuates with the change of cavitation pressure under different roughness conditions (Fig. 13). However, the change is small, with the average value of approximately 80%.

Meanwhile, the area ratios of the optimal dimple (66.0% and 71.6%) can be obtained under different operating parameters, and its change law varies with the roughness parameters. The area ratio remains constant at 71.6% when *σ* = 1.5 μm. The area ratio increases from 66.0% to 76.1% under different surface pattern parameters with the increase in cavitation pressure. Furthermore, when *γ* = 1, the cavitation pressure corresponding to the jump point has the maximum value, while *γ* = 1/3, the cavitation pressure corresponding to the jump point has a minimum value.

(3) Variation of optimal depth

**Figure 14:**
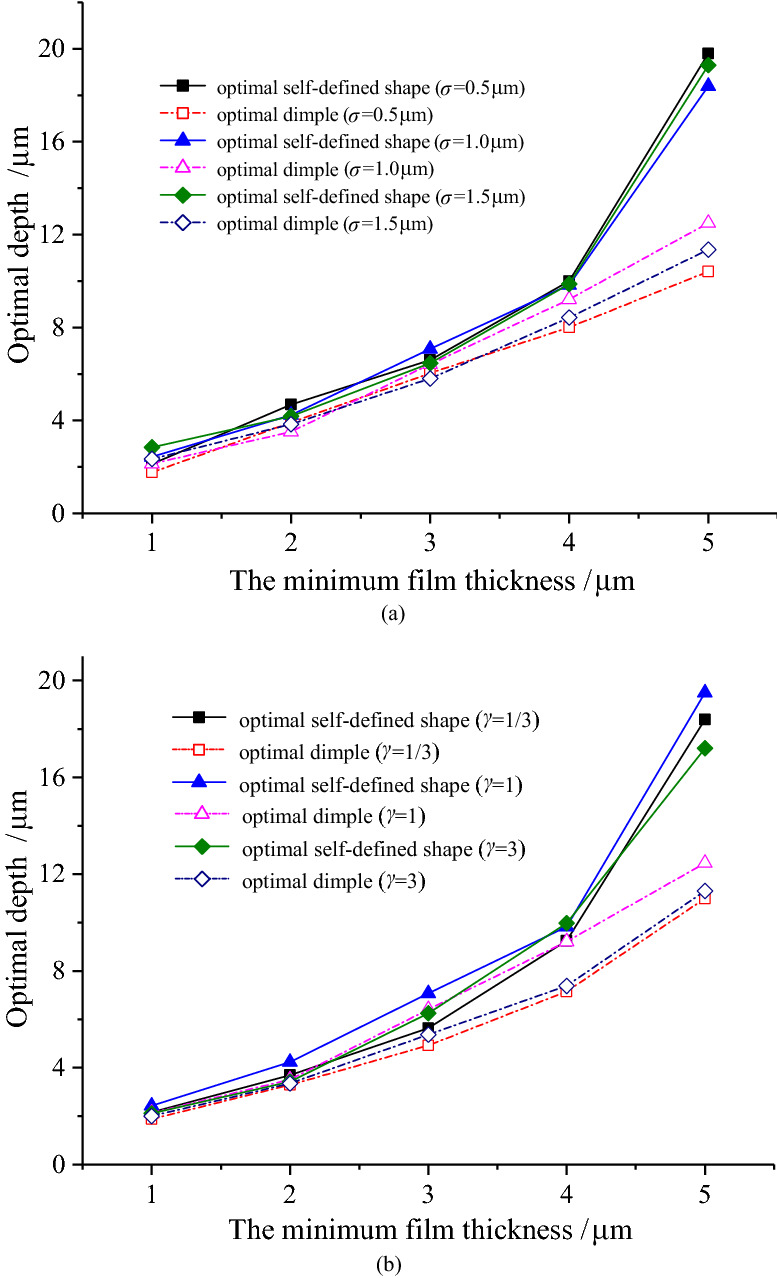
Variation of the optimal depth with the minimum film thickness.

In the case of the minimum film thickness increases, the optimal depth gradually increases for optimal self-defined shape and optimal dimple as well, as depicted in Fig. 14. The depth of the optimal dimple is slightly less than that of the optimal self-defined shape when the minimum film thickness is small. Under the condition of the minimum film thickness increases, the discrepancy between depths of optimal self-defined shape and optimal dimple also gradually expands, and the maximum value reaches 9 μm at *h*_0_ = 5 μm. However, the optimal depth has little difference under various roughness parameters for the same minimum film thickness, indicating that the roughness parameter has little effect on the optimal depth. In addition, the optimal depth at *γ* = 1 is larger than that at other values.

**Figure 15:**
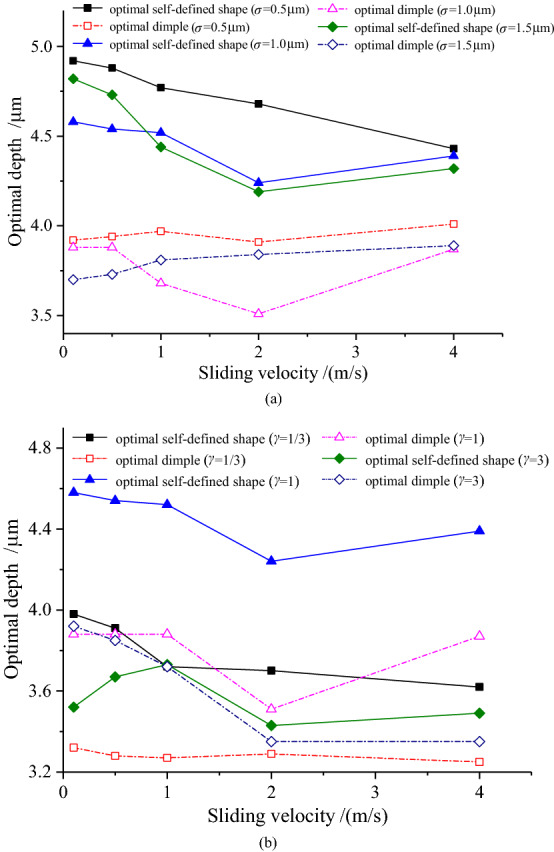
Variation of the optimal depth with sliding velocity.

**Figure 16:**
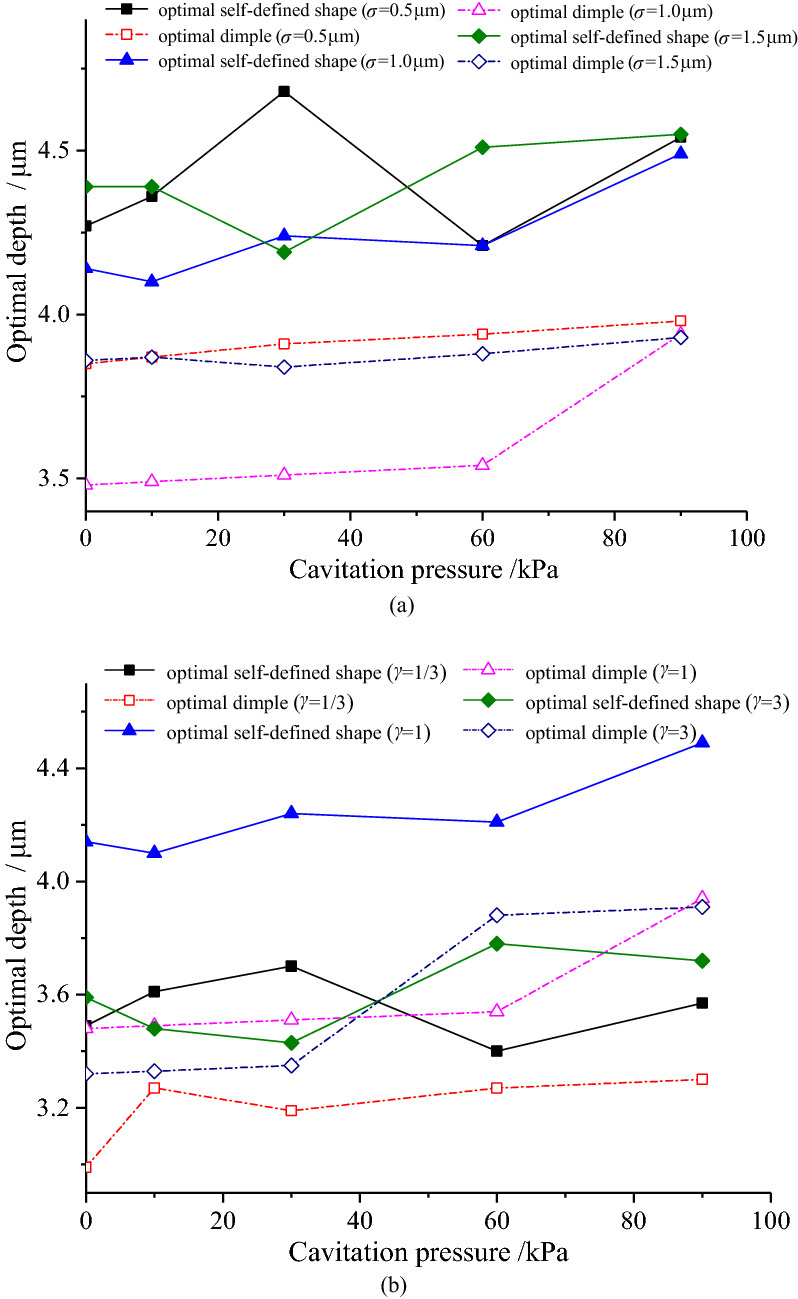
Variation of the optimal depth with cavitation.

Figure 15–16 demonstrate that the depths of the optimal self-defined shape and the optimal dimple slightly change (within 1 μm) with the increase in the sliding velocity and cavitation pressure, indicating that either the sliding velocity or cavitation pressure has limited effect on the optimal depth. In addition, the depth of the optimal self-defined shape is larger than that of the optimal dimple under various roughness parameters, sliding velocities, and cavitation pressures. Nevertheless, the difference is not significant, demonstrating that the roughness parameters have little effect on the optimal depth.

## Conclusions

In the process of solving the rough contact model, the average Reynolds equation is combined, and the optimal texture shape considering the surface roughness is obtained with the genetic algorithm method. In order to properly consider the cavitation influence, the JFO mass-conservation boundary condition is used. The influences of surface roughness, surface pattern parameter, and operating parameters on the friction coefficient, area ratio, and optimal depth for the optimal self-defined shape and the optimal dimple are analyzed. The following is a summary of the findings:

1. A considerable friction coefficient reduction will be obtained throughout shape optimization when taking the roughness condition into account, and the influence of roughness parameter cannot be ignored.
2. The area ratio of the optimal dimple is always smaller than that of the optimal self-defined shape, which increases with the increase in the combined RMS, and when the surface morphology parameters, minimum film thickness and sliding velocity increase, the variation trend of friction coefficient is to decrease first and then increase.
3. The depth of the optimal self-defined shape almost unchanged with the variation of surface roughness, sliding velocity, and cavitation pressure except for the case of large oil minimum film thickness.
4. Roughness parameters have a greater influence on the optimal dimple than that on the optimal custom shape under different sliding velocities.

## Supplementary Information

Supplementary Information.

## Data Availability

The data that support the findings of this study are available from the corresponding author upon reasonable request.

## References

[1] Gropper D, Wang L, Harvey TJ (2016). Hydrodynamic lubrication of textured surfaces: A review of modeling techniques and key findings. Tribol. Int..

[2] Malik S, Kakoty S (2014). Analysis of dimple textured parallel and inclined slider bearing. Proc. Inst. Mech. Eng. Part J..

[3] Li D, Yang X, Lu C, Cheng J, Wang S, Wang Y (2019). Tribological characteristics of a cemented carbide friction surface with chevron pattern micro-texture based on different texture density. Tribol. Int..

[4] Zum G, Wahl R, Wauthier K (2008). Experimental study of the effect of microtexturing on oil lubricated ceramic/steel friction pairs. Wear.

[5] Wang W, Liu Z, Chen D, Xie Z, Song J (2021). Influence of different surface texture parameters on the contact performance of piston ring-sleeve friction pair of hydraulic cylinders. Adv. Mater. Sci. Eng..

[6] Wang H, Zhu H, Zhou Y, Yang H (2015). Experimental study on the friction characteristics of textured steel surface with ring-shaped pits under lubricated sliding conditions. Tribol. Trans..

[7] Krupka I, Hartl M (2007). Experimental Study of microtextured surfaces operating under thin-Film EHD lubrication conditions. J. Tribol..

[8] Ryk G, Kligerman Y, Etsion I (2002). Experimental investigation of laser surface texturing for reciprocating automotive components. Tribol. Trans..

[9] Yan D, Qu N, Li H, Wang X (2010). Significance of dimple parameters on the friction of sliding surfaces investigated by orthogonal experiments. Tribol. Trans..

[10] Wang H, Li Y, Zhu H (2016). Experimental study on the tribological performance of fractal-like textured surface under mixed lubrication conditions. Surf. Coat. Technol..

[11] Khonsari M, Qiu Y (2011). Performance analysis of full-film textured surfaces with consideration of roughness effects. J. Tribol..

[12] Yu H, Deng H, Huang W, Wang X (2011). The effect of dimple shapes on friction of parallel surfaces. Proc. Inst. Mech. Eng. Part J.

[13] Yu H, Huang W, Wang X (2013). Dimple patterns design for different circumstances. Lubr. Sci..

[14] Ahmed A, Masjuki H, Varman M, Kalam M, Habibullah M, Mahmud K (2016). An overview of geometrical parameters of surface texturing for piston/cylinder assembly and mechanical seals. Meccanica.

[15] Zhan X, Yi P, Liu Y, Xiao P, Zhu X, Ma J (2020). Effects of single- and multi-shape laser-textured surfaces on tribological properties under dry friction. Proc. Inst. Mech. Eng. Part C.

[16] Uddin MS, Ibatan T, Shankar S (2017). Influence of surface texture shape, geometry and orientation on hydrodynamic lubrication performance of plane-to-plane slider surfaces. Lubr. Sci..

[17] Yu H, Wang X, Zhou F (2010). Geometric shape effects of surface texture on the generation of hydrodynamic pressure between conformal contacting surfaces. Tribol. Lett..

[18] Qiu M, Delic A, Raeymaekers B (2012). The effect of texture shape on the load-carrying capacity of gas-lubricated parallel slider bearings. Tribol. Lett..

[19] Wu Q, Zhang H, Zhao W, Zhao X (2020). Shape optimum design by basis vector method considering partial shape dependence. Appl. Sci..

[20] Shen C, Khonsari M (2015). Numerical optimization of texture shape for parallel surfaces under unidirectional and bidirectional sliding. Tribol. Int..

[21] Yoshida I, Tsukada T (2006). Uncertainty of wavelength limitation due to stylus tip radius for engineering surface texture based on wavelength and amplitude by FFT. Wear.

[22] Uchidate M, Yanagi K, Yoshida I (2011). Generation of 3D random topography datasets with periodic boundaries for surface metrology algorithms and measurement standards. Wear.

[23] Yang Z, Ding X, Liu J, Zhang F (2019). Effect of the finite size of generated rough surfaces on the percolation threshold. Proc. Inst. Mech. Eng. Part C.

[24] Ma C, Duan Y, Yu B, Sun J, Tu Q (2017). The comprehensive effect of surface texture and roughness under hydrodynamic and mixed lubrication conditions. Proc. Inst. Mech. Eng. Part J..

[25] Zhou Y, Zhu H, Zhang W, Zuo X, Li Y, Yang J (2015). Influence of surface roughness on the friction property of textured surface. Adv. Mech. Eng..

[26] Ma G, Jiang L, Huang W (2020). Lubrication properties of textured polydimethysiloxane surfaces with different roughness. J. Xi'an Jiaotong Univ..

[27] Xie Y, Li Y, Suo S, Liu X, Li J, Wang Y (2013). A mass-conservative average flow model based on finite element method for complex textured surfaces. Sci. China Phys. Mech. Astron..

[28] Patir N, Cheng HS (1978). An average flow model for determining effects of three-dimensional roughness on partial hydrodynamic lubrication. J. Lubr. Technol..

[29] Wu C, Zheng L (1989). An average reynolds equation for partial film lubrication with a contact factor. J. Tribol..

[30] Patir N, Cheng HS (1979). Application of the average flow model to lubrication between rough sliding surfaces. J. Lubr. Technol..

[31] Elrod, H. G. & Adams, M. A computer programme for cavitation. In *Processing of the First Leeds-Lyon Symposium on Cavitation and Related Phenomena in Lubrication* 37–41 (Mechanical Engineering Publication, USA, 1974).

[32] Ausas R, Ragot P, Leiva J, Jai M, Bayada G, Buscaglia G (2007). The impact of the cavitation model in the analysis of microtextured lubricated journal bearings. J. Tribol..

[33] Greenwood JA, Tripp JH (1971). The contact of two nominally flat rough surfaces. Proc. Inst. Mech. Eng..

[34] Hu Y, Cheng H, Arai T, Kobayashi Y, Aoyama S (1994). Numerical simulation of piston ring in mixed lubrication—a nonaxisymmetrical analysis. J. Tribol..

