# Supplementary material for: On the optimal texture shape with the consideration of surface roughness
Source: Sci Rep. 2022 Sep 1;12:14878. doi: 10.1038/s41598-022-19094-8 (PMC9437064; doi:10.1038/s41598-022-19094-8)
Supplement: Supplementary file 1 — Supplementary Information. [file 41598_2022_19094_MOESM1_ESM.docx]

# Appendix

# Notation

|  | pressure flow factors in the *x* and *z*  directions |
| --- | --- |
|  | shear flow factor |
|  | shear stress factor |
|  | shear stress correction factor |
| $\overline{p}$ | mean pressure of the fluid |
| $\overline{h_{T}}$ | average gap that equals the expected or mean value of $h_{T}$ |
| $h_{T}$ | local film thickness (μm) |
| $\mu$ | dynamic viscosity (Pa·s) |
| $\mu_{f}$ | friction coefficient of the contact asperity |
| $u$ | sliding velocity (m/s) |
| $h_{0}$ | minimum nominal film thickness (μm) |
| $h_{p}$ | dimple radio (μm) |
| $p$ | fluid pressure (Pa) |
| $p_{cav}$ | cavitation pressure (kPa) |
| $\theta$ | film content |
| $E^{\text{*}}$ | equivalent elastic modulus |
| $K^{\text{，}}$ | constant $K^{\text{，}}\text{=}5.319\times10^{10}\times\sigma^{2.5}$ |
| $B$ | constant $B\text{=}4.406\times10^{-5}$ |
| $\Omega$ | constant $\Omega\text{=}4.0$ |
| $W$ | load-carrying capacity (N) |
| $W_{avg}$ | average pressure of the unit cell (Pa) |
| $\sigma$ | combined RMS roughness |
